# Diastolic preparation for left ventricular ejection - A marker of inefficiency of the failing heart

**DOI:** 10.1186/1532-429X-13-S1-O84

**Published:** 2011-02-02

**Authors:** Jonatan Eriksson, Petter Dyverfeldt, Jan Engvall, Ann F Bolger, Tino Ebbers, Carl Johan Carlhäll

**Affiliations:** 1 Center for Medical Image Science and Visualization (CMIV), Linköping University, Linköping, Sweden; 2 University of California San Francisco, San Francisco, CA, USA

## Background

Heart failure represents the final stage of various cardiac disorders. In dilated and hypocontractile left ventricles (LV), alterations in intraventricular blood flow patterns have been observed [1,2]. Whether or not these alterations reflect LV pumping efficiency is not completely understood.

## Hypothesis

We hypothesized that diastolic flow in the normal LV would prepare an effective systolic ejection by virtue of increased end-diastolic (ED) kinetic energy (KE) of the ejected blood. Further, we hypothesized that this pre-systolic preparation for LV ejection would be impaired in the failing LV.

## Methods

**Figure 1:**
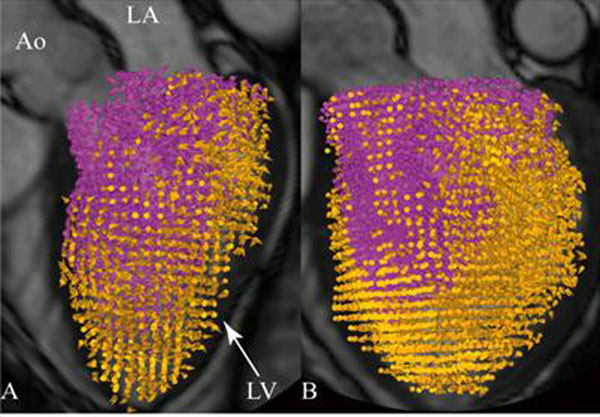
Pathline visualization at end-diastole of LV ejecting blood (purple color) and non-ejecting (orange color), respectively. A) Healthy; B) Patient. Semi-transparent three-camber image provides anatomical orientation. Ao, aorta; LA, left atrium; LV, left ventricle.

In eight dilated cardiomyopathy (DCM) patients (4 female, 51±13 years [mean±SD]) and twelve healthy (H) subjects (5 female, 44±17 years), 4D phase-contrast CMR velocity data and morphological images were acquired at 1.5T (Philips Achieva). A previously validated method was used for the analysis (3): The LV endocardium was segmented (http://segment.heiberg.se) from short-axis images at ED and end-systole (ES). Pathlines were emitted from the LV ED blood volume and traced forward and backward in time until ES. Accordingly, the ED blood volume could be automatically separated into ejecting blood and non-ejecting blood (Figure 1). The KE was calculated over the cardiac cycle for these flow components based on the volume occupied by each pathline, its velocity, and blood density.

## Results

**Figure 2:**
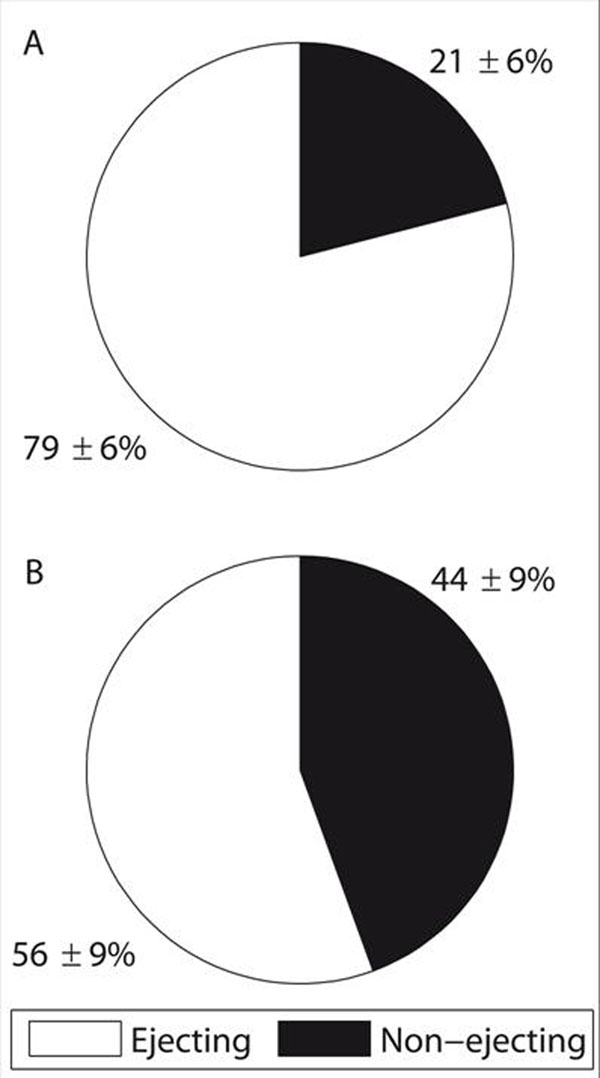
Illustration of the “KE of the ejecting blood”/”KE of the total end-diastolic blood” fraction at end-diastole A) Healthy; B) DCM patients

The LV ED diameter was larger and LV ejection fraction was smaller in DCM compared to H (61±6 vs 46±4 mm, and 42±5 vs 61±3 %, respectively, both p<0.001). At ED, the “KE of the non-ejecting blood” was higher in DCM compared to H (0.8±0.4 vs 0.2±0.1 mJ, p<0.001), whereas there was no difference in the “KE of the ejecting blood” between the groups (0.9±0.3 vs 0.7±0.2 mJ, NS). At ED, the “KE of the ejecting blood”/”KE of the total ED blood”-ratio was lower in DCM compared to H (p<0.001) (Figure 2).

## Conclusion

Diastolic flow is quantitatively altered in dilated and hypocontractile LVs. At ED, the “KE of the ejecting blood” represented a significantly smaller fraction of the “KE of the total ED blood” in failing LVs compared to normal LVs. Loss of this “energetic preparation” for ensuing ejection may contribute to the inefficiency of the failing LV. Medical or interventional strategies that preserve physiological flow in the diastolic LV may also improve systolic performance.
